# Gap between availability and utilization of cardiac donors: analysis of determining factors in Ecuador

**DOI:** 10.47487/apcyccv.v6i4.530

**Published:** 2025-12-29

**Authors:** Guillermo Solórzano Suárez, Paola Morejón Barragán, José Miguel Jáuregui Solórzano, Mónica Gilbert Orús

**Affiliations:** 1 Clínica Guayaquil, Guayaquil, Ecuador. Clínica Guayaquil Guayaquil Ecuador; 2 Servicio de Cardiología, Insuficiencia Cardiaca Avanzada, Soporte Circulatorio Mecánico y Trasplante Cardiaco, Clínica Guayaquil, Guayaquil, Ecuador. Servicio de Cardiología, Insuficiencia Cardiaca Avanzada Soporte Circulatorio Mecánico y Trasplante Cardiaco Clínica Guayaquil Guayaquil Ecuador; 3 Servicio de Medicina Crítica, coordinador hospitalario de trasplantes, Clínica Guayaquil, Guayaquil, Ecuador. Servicio de Medicina Crítica coordinador hospitalario de trasplantes Clínica Guayaquil Guayaquil Ecuador; 4 Servicio de Cirugía Cardiovascular y Trasplante Cardiaco, Clínica Guayaquil, Guayaquil, Ecuador. Servicio de Cirugía Cardiovascular y Trasplante Cardiaco Clínica Guayaquil Guayaquil Ecuador

**Keywords:** Transplantation, Tissue and Organ Procurement, Heart Transplantation, Health Care Rationing, Trasplante, Obtención de Tejidos y Órganos, Trasplante de Corazón, Asignación de Recursos para la Atención de Salud

## Abstract

**Objectives.:**

To analyse factors associated with the rejection of heart donor offers referred to Clínica Guayaquil and to compare these findings with national data from the Instituto Nacional de Donación y Trasplante de Órganos, Tejidos y Células, in order to identify trends influencing organ acceptance.

**Materials and Methods.:**

We conducted an observational, retrospective study of heart donor offers received between September 2021 and July 2025. Demographic, clinical, anthropometric, and logistical variables were extracted from the institutional database and the National Information System for Donation and Transplantation (SINIDOT). Reasons for organ rejection were classified into eight predefined categories. Univariate and multivariate analyses were performed to identify factors associated with donor acceptance or rejection.

**Results.:**

A total of 196 heart donor offers were received, of which 75% were rejected. Accepted donor organs were from younger donors, were more frequently male, and had higher predicted heart mass (PHM). Traumatic brain injury was the leading cause of death (49.5%). The most common reasons for rejection were classification as a non-standard risk donor (39.5%), logistical constraints (30.6%), and blood group incompatibility (15.6%). In multivariate analyses, older donor age and origin outside Guayaquil were associated with higher rejection rates, whereas male sex and higher PHM were associated with increased acceptance. Logistical problems rose from 0% in 2021 to more than 40% in 2024-2025, largely driven by limited availability of air transport.

**Conclusions.:**

The high rate of donor heart rejection reflects substantial underutilisation of potentially viable organs. Strengthening transport logistics and broadening donor acceptance criteria could increase graft utilisation, reduce waiting-list mortality, and improve the overall efficiency of national heart transplantation programmes.

## RESUMEN

**Objetivos.:**

Analizar los factores asociados al rechazo de ofertas de donantes cardíacos dirigidas a la Clínica Guayaquil y compararlos con datos nacionales del Instituto Nacional de Donación y Trasplante de Órganos, Tejidos y Células, para identificar tendencias que influyen en la aceptación de órganos.

**Materiales y métodos.:**

Estudio observacional y retrospectivo de ofertas cardíacas recibidas entre septiembre de 2021 y julio de 2025. Se recopilaron variables demográficas, clínicas, antropométricas y logísticas de la base institucional y del Sistema Informático Nacional de Donación y Trasplante (SINIDOT). Las causas de rechazo se clasificaron en ocho categorías. Se aplicaron análisis univariados y multivariados para identificar asociaciones.

**Resultados.:**

Se recibieron 196 ofertas; el 75% fueron rechazadas. Los donantes aceptados fueron más jóvenes, mayoritariamente masculinos y con mayor masa cardíaca predicha (PHM, por sus siglas en inglés, Predicted Heart Mass). El traumatismo craneoencefálico fue la principal causa de muerte (49.5%). Las razones más frecuentes de rechazo fueron «donante de riesgo no estándar» (39,5%), problemas logísticos (30,6%) e incompatibilidad sanguínea (15,6%). En el análisis multivariado, la edad avanzada y la procedencia fuera de Guayaquil se asociaron con un mayor rechazo; el sexo masculino y un PHM elevado, con un menor rechazo. Los problemas logísticos aumentaron del 0% en 2021 a más del 40% en 2024-2025, principalmente por falta de transporte aéreo.

**Conclusiones.:**

La alta tasa de rechazo refleja una subutilización de órganos potencialmente viables. Optimizar la logística de transporte y ampliar criterios de aceptación podría aumentar la utilización de órganos, reducir la mortalidad en la lista de espera y mejorar la eficiencia de los programas de trasplante cardíaco a nivel nacional.

## Introduction

End-stage heart failure remains one of the leading causes of morbidity and mortality worldwide, and heart transplantation continues to represent the therapeutic option that offers the greatest survival benefit and best quality of life for these patients ^1^. However, the gap between demand and the availability of donated organs remains wide, and donor scarcity is driven not only by low notification rates but also by the high proportion of organs discarded during the selection process and procurement logistics ^2^. The Ecuadorian setting mirrors this trend. According to accountability reports from the National Institute for Organ, Tissue and Cell Donation and Transplantation (INDOT, in Spanish), the national rate of effective donors increased from 2.76 donors per million inhabitants in 2021 to 5.3 donors per million inhabitants in 2022 ^3^^-^^5^. Nevertheless, in 2024, this rate declined to 3.92 donors per million inhabitants, corresponding to 72 effective donors reported to the Institute ^6^. During the same year, 17 heart transplants were performed nationwide ^6^. These figures indicate sustained activity in donor identification but also highlight that the procurement of viable hearts for transplantation remains substantially below clinical needs.

Clínica Guayaquil has been one of the two accredited and authorised centres for heart transplantation in Ecuador since 2021. In 2024, it accounted for 70.59% of all heart transplant procedures performed nationwide ^6^ and systematically receives cardiac offers notified by the National Institute. Analysing the reasons for rejection of these offers at this institution represents an opportunity to identify the main factors limiting organ utilisation at the national level. Understanding these factors is essential for designing strategies to optimise organ acceptance, reduce waiting-list mortality, and maximise the efficiency of resources allocated to procurement. Despite the relevance of this issue, published evidence from Latin America remains scarce, and most evaluation protocols continue to rely on studies conducted in North America or Europe ^7^^-^^11^.

The aim of this study is to analyse the factors associated with the rejection of cardiac donor offers directed to Clínica Guayaquil, contrasting them with consolidated data reported by the National Institute during the same period. The primary objective is to identify trends and variables influencing organ acceptance and, ultimately, the number of heart transplants performed in the country. The findings are intended to provide local evidence to inform updates to donor selection and acceptance algorithms, as well as to optimise and strengthen interhospital logistical coordination.

## Materials and methods

### Study design

An observational, retrospective study was conducted of cardiac donor offers received by the Heart Transplant Unit of Clínica Guayaquil. The analysis period spanned from September 1, 2021, to July 30, 2025. Data were obtained from the institutional database of Clínica Guayaquil, as well as from the centre’s transplant activity records accessed through the National Donation and Transplant Information System (SINIDOT, in Spanish), which registers all cardiac donor offers notified by the INDOT. The SINIDOT-generated report includes demographic, clinical, and logistical characteristics of each donor, together with the final decision regarding organ acceptance or rejection, including the corresponding justification. In addition, official INDOT accountability reports for the years 2021, 2022, 2023, and 2024 were reviewed, as these provide national-level consolidated statistics on organ donation and transplantation activities ^3^^,^^5^^,^^6^^,^^12^.

### Study population

The study population included all donors notified for cardiac organ offers by INDOT to Clínica Guayaquil through SINIDOT during the study period. The Heart Transplant Unit of Clínica Guayaquil receives these offers exclusively when the organ is allocated to one of the active recipients on its institutional waiting list. No additional exclusion criteria were applied, and all registered cases were analysed.

### Variables 

Demographic variables (age, sex, blood group, geographic origin, and type of institution [public vs. private]), anthropometric variables (weight, height, and predicted heart mass [PHM]), and clinical variables (cause of death; relevant medical history including hypertension, diabetes mellitus, known heart disease, and cardiopulmonary resuscitation; history of smoking, cocaine or methamphetamine use; and vasoactive support) were analysed. The outcome variables included the final decision (accepted or rejected) and the specific reason for rejection. Reasons for rejection were grouped into eight mutually exclusive categories: (1) blood group incompatibility, defined as ABO mismatch between the donor and listed recipients; (2) non-standard risk donor, defined as donors with characteristics outside conventional acceptance criteria, including advanced age, multiple comorbidities, active infections, neoplasms, and clinical conditions associated with increased perioperative risk ^13^; (3) anthropometric factors, defined as a significant mismatch between donor and recipient predicted heart mass (>20-30% difference) ^8^; (4) donor heart disease, including structural or functional cardiac pathology; (5) logistical issues, including limitations in transport, coordination, or resource availability; (6) inactive recipient, defined as temporary unavailability due to medical condition; (7) positive direct crossmatch; and (8) other causes not classifiable within the preceding categories.

As a complementary reference, the low-dose criterion proposed by the International Society for Heart and Lung Transplantation was considered, which defines acceptable vasoactive support as norepinephrine ≤0.1 μg/kg/min without the use of additional inotropes ^(^^8^.

### Procedures and interventions

This study did not involve direct clinical interventions. Each cardiac offer received through SINIDOT was evaluated, and all variables described above were analysed. The final decision regarding acceptance or rejection was made in accordance with the institutional protocol and recorded together with the corresponding justification.

### Ethical aspects

The study was based on the retrospective analysis of routinely collected institutional data, with no intervention involving patients. Data confidentiality was ensured in accordance with institutional regulations through data coding, and the ethical principles of the Declaration of Helsinki for medical research were upheld. Given the observational and retrospective nature of the study, specific informed consent was not required. The study was approved by the hospital ethics committee.

### Statistical analysis

A descriptive analysis of the variables was performed. Continuous variables are presented as mean ± one standard deviation and were compared between accepted and rejected groups using the Student’s t test for independent samples, after verifying approximate normality and homogeneity of variances. A p-value <0.05 was considered statistically significant. Categorical variables are presented as absolute and relative frequencies and were compared between groups using Pearson’s chi-square test.

The association between each variable and donor rejection was assessed using univariable binary logistic regression, estimating odds ratios (ORs) with 95% confidence intervals (95% CIs), using the most frequent or clinically defined category as the reference. Variables with p<0.05 were included in a multivariable model. Data were collected in Google Sheets and processed using Python and DataTab.

## Results

A total of 196 cardiac donor offers were analysed, of which 49 (25.0%) were accepted and 147 (75.0%) were rejected. The mean donor age was 37.1 ± 12.4 years, with a predominance of male donors (n=136; 69.4%). Regarding blood group distribution, group O was the most frequent (n=147; 75.0%), followed by A (n=33; 16.8%) and B (n=16; 8.2%); no donors with blood group AB were recorded. Mean donor weight was 72.7 ± 10.0 kg, mean height 1.66 ± 0.1 m, and mean PHM 165.1 ± 24.2 g. With respect to haemodynamic management, most donors required pharmacological support: 64.8% (n=127) received one vasopressor and/or inotropic agent, 28.6% (n=56) received two agents, and 3.6% (n=7) received three or more agents, whereas only 3.1% (n=6) required no vasoactive drugs.

The leading cause of death among offered donors was severe traumatic brain injury, identified in 97 cases (49.5%), followed by haemorrhagic stroke in 75 cases (38.3%) and ischaemic stroke in 22 cases (11.2%). Less frequent causes included brain tumour and other aetiologies, with one case each.

In the comparative analysis between accepted and rejected offers, accepted donors were significantly younger and more frequently male. Among anthropometric variables, only height and PHM showed statistically significant differences, both being higher in the accepted group. No differences were observed in the distribution of causes of death; however, severe traumatic brain injury was more frequent among accepted donors. Likewise, blood group O showed a significantly higher prevalence in this group (Table 1).

**Table 1:** Baseline characteristics according to acceptance or rejection status of the donor offer.

Status	Accepted n=49	Rejected n=147	p value
Baseline characteristics			
Age (years)	31.98 ± 9.52	38.86 ± 12.81	<0.001
Male sex	43 (87.8)	93 (63.3)	0.002
Anthropometry			
Weight (kg)	73.49 ± 10.09	72.44 ± 9.96	0.529
Height (m)	1.69 ± 0.09	1.65 ± 0.08	0.028
Body surface area (m^2^)	1.83 ± 0.16	1.80 ± 0.15	0.141
Body mass index (kg/m²)	25.90 ± 3.23	26.58 ± 3.48	0.217
Predicted heart mass (g)	173.76 ± 23.01	162.22 ± 24.00	0.003
Cause of death			
Severe TBI	30 (61.2)	67 (45.6)	0.083
Haemorrhagic stroke	16 (32.7)	59 (40.1)	0.445
Ischaemic stroke	2 (4.1)	20 (13.6)	0.073
Brain tumour	0 (0.0)	1 (0.7)	1.000
Other	1 (2.0)	0 (0.0)	0.250
Blood group			
O	43 (87.8)	104 (70.7)	0.028
A	4 (8.2)	29 (19.7)	0.077
B	2 (4.1)	14 (9.5)	0.366
AB	0 (0.0)	0 (0.0)	-
Medical history			
Hypertension	8 (16.3)	26 (17.7)	1.000
Diabetes mellitus	1 (2.0)	7 (4.8)	0.682
Cardiopulmonary resuscitation	2 (4.1)	9 (6.1)	0.734
Smoking	1 (2.0)	15 (10.2)	0.078
Cocaine or methamphetamine use	1 (2.0)	1 (0.7)	0.438
Known heart disease	0 (0.0)	7 (4.8)	0.196
Vasoactive support			
Number of agents			
None	2 (4.1)	4 (2.7)	0.641
1	23 (46.9)	104 (70.7)	0.004
2	19 (38.8)	37 (25.2)	0.100
≥3	5 (10.2)	2 (1.4)	0.015
Agents			
Norepinephrine	45 (91.8)	142 (96.6)	0.231
Vasopressin	18 (36.7)	18 (12.2)	<0.001
Dobutamine	3 (6.1)	2 (1.4)	0.101
Dopamine	9 (18.4)	12 (8.2)	0.083
Epinephrine	0 (0.0)	9 (6.1)	0.115
ISHLT low-dose norepinephrine criterion			
≤0,1 mcg/kg/min	28 (57.1)	62 (42.2)	0.098
>0,1 mcg/kg/min	17 (34.7)	80 (54.4)	0.026

Data are presented as n (%) or mean ± standard deviation.

ISHLT: International Society for Heart and Lung Transplantation. TBI: traumatic brain injury.

Regarding haemodynamic support, the use of a single vasoactive agent was more common among rejected donors, whereas the use of three or more agents, as well as vasopressin, was more frequent among accepted donors (Table 1**)**. Administration of norepinephrine at doses >0.1 μg/kg/min was more common in rejected offers. Notably, donor cardiac function could not be included as an analytical variable, as this information is not captured in the standardised INDOT notification form ^14^, precluding its systematic availability across all evaluated offers.

**Figure 1:**
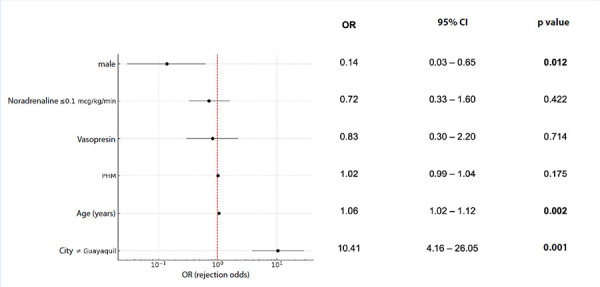
Total number of cardiac donor offers made to Clínica Guayaquil per year and temporal trends in acceptance rates

Temporal analysis showed a progressive increase in the number of notifications, peaking in 2023 with 72 offers (36.7%), followed by 2024 with 55 (28.1%) and 32 offers (16.3%) recorded up to July 2025. In contrast, 2021 and 2022 showed substantially lower activity, with 11 (5.6%) and 26 notifications (13.3%), respectively, attributable to the residual impact of the COVID-19 pandemic and the fact that the heart transplant programme formally began in November 2021, limiting the number of offers during that initial period. Acceptance rates varied considerably over time, with 2022 showing the highest acceptance rate (38.5%) and 2025 the lowest (18.8%) (Figure 1).

In terms of geographic origin, Quito was the main source of offers (81 cases; 41.3%), followed by Guayaquil (73 cases; 37.2%) and Cuenca (19 cases; 9.7%). Ambato and Latacunga each contributed seven offers (3.6% each), while other localities accounted for the remaining 4.6%. By type of institution, public hospitals predominated, contributing 146 offers (74.5%), compared with 50 offers (25.5%) from private centres.

Analysis of the 147 documented rejections showed that the predominant cause was classification as a non-standard risk donor, accounting for 58 cases (39.5%). The second most frequent cause was logistical issues, responsible for 45 cases (30.6%), of which 37 were related to lack of air transport availability and five to failures in interinstitutional coordination and communication. Table 2 details the causes of rejection and the characteristics of non-standard risk donors. Notably, among donors rejected due to age, 13 (50.0%) were aged 50-54 years and 13 (50.0%) were aged 55 years or older, with a maximum reported age of 78 years.

**Table 2:** Causes of rejection of notified cardiac donor offers.

Cause of rejection	n	(%)
Non-standard risk donor	58	(39.5)
Age	26	(44.8)
50 - 55	13	(50.0)
>55	13	(50.0)
Estimated cold ischaemia time >4 h	17	(29.3)
Medical or toxicological history	17	(29.3)
Infection*	8	(13.8)
Unclassified central nervous system tumour†	1	(1.7)
Other	2	(3.4)
Logistics	45	(30.6)
Unavailability of air transport	37	(82.2)
Interinstitutional coordination or communication failures	5	(11.1)
Other	3	(6.7)
ABO incompatibility	23	(15.6)
Predicted heart mass mismatch	12	(8.2)
Pre-existing heart disease	5	(3.4)
Inactive recipient	2	(1.4)
Positive direct crossmatch	1	(0.7)
Irreversible donor cardiac arrest	1	(0.7)

* Seven donors had bacteraemia with less than 48 h of targeted antibiotic therapy against identified pathogens; three met sepsis criteria. Additionally, three donors had associated pneumonia, one had a urinary tract infection, and one donor tested positive for HBsA.

† Central nervous system tumour with prior surgery, without histopathology; considered high grade (>10% transmission risk).

Among cardiac pathologies leading to non-acceptance, the following were identified: one donor (20.0%) with a history of cardiac surgery, one (20.0%) with congenital heart disease and pulmonary hypertension, and three donors (60.0%) with moderate or severe ventricular dysfunction and regional wall motion abnormalities.

**Figure 2:**
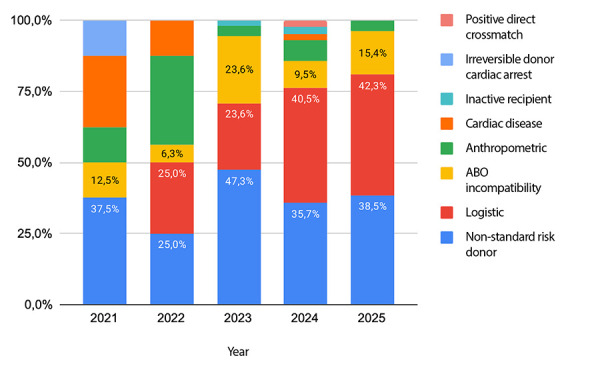
Causes of rejection of cardiac donor offers by year. Data cover the period from September 2021 to July 2025

Annual analysis of rejection causes showed that non-standard risk donors consistently remained one of the main reasons for discard across all periods, with proportions ranging from 25.0% to 47.3%. Logistical issues increased progressively from 0% in 2021 to over 40% in 2024 and 2025, becoming the predominant cause in those years (Figure 2).

Rejection causes were classified as modifiable factors (logistics-related) and non-modifiable factors (inherent to the donor or to the clinical condition of recipients on the waiting list). Overall, 69.4% (n=102) of donor heart rejections were due to non-modifiable factors, while 30.6% (n=45) were attributable to modifiable logistical causes. Among non-modifiable factors, the non-standard risk donor category was the most frequent, followed by blood group incompatibility and anthropometric mismatches (Table 2).

In univariable analysis, older age (p=0.001) and origin outside Guayaquil (p=0.001) were associated with higher odds of rejection, whereas male sex (p=0.028), higher PHM (p=0.043), use of vasopressin (p=0.021), and norepinephrine support ≤0.1 μg/kg/min (p=0.049) were associated with lower odds of rejection (Table 3).

**Table 3:** Univariable analysis of factors associated with donor rejection

Variable	Category	OR (95% CI)	p value
City	Other vs. Guayaquil	9.51 (4.48-20.16)	<0.001
Vasopressin	Yes	0.24 (0.11-0.52)	<0.001
Age	Per unit	1.05 (1.02-1.08)	0.001
Sex	Male	0.24 (0.10-0.60)	0.002
PHM	Per unit	0.98 (0.97-0.99)	0.005
Norepinephrine	≤0.1 vs. >0.1 mcg/kg/min	0.47 (0.24-0.94)	0.032
Norepinephrine	0 vs. >0.1 mcg/kg/min	0.27 (0.07-1.09)	0.066
Dopamine	Yes	0.40 (0.16-1.01)	0.051
Dobutamine	Yes	0.21 (0.03-1.31)	0.094
Hypertension	Yes	1.10 (0.46-2.62)	0.828
Diabetes mellitus	Yes	2.40 (0.29-20.01)	0.418
History of smoking	Yes	5.46 (0.70-42.41)	0.105
Cocaine or methamphetamine use	Yes	0.33 (0.02-5.36)	0.435
Cardiac arrest	Yes	1.67 (0.35-8.08)	0.523
Cause of death	TBI vs. haemorrhagic stroke	0.61 (0.30-1.22)	0.161
Cause of death	Other causes vs. haemorrhagic stroke	1.90 (0.50-7.18)	0.345

OR: odds ratio. CI: confidence interval. TBI: traumatic brain injury. PHM: predicted heart mass.

**Figure 3:**
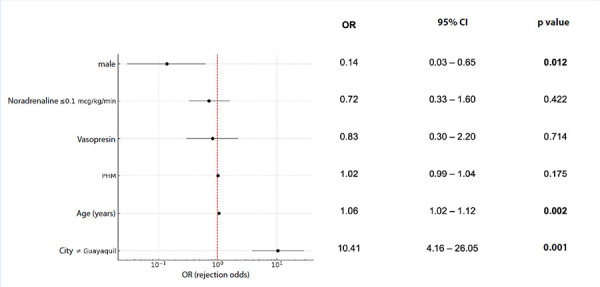
Forest plot of the multivariable model for factors associated with cardiac donor rejection. 95% CI: 95% confidence Interval, PHM: predicted heart mass.

In the multivariable analysis (Figure 3), origin outside Guayaquil remained independently associated with a significant increase in rejection odds (OR: 10.41; 95% CI: 4.16-26.05; p<0.001), whereas male sex was independently associated with a significant reduction in this risk (OR: 0.14; 95% CI: 0.03-0.65; p=0.012). Donor age was also positively associated with rejection (OR: 1.06 per year; 95% CI: 1.02-1.10; p=0.002). No significant associations were observed for PHM, vasopressin use, or norepinephrine support ≤0.1 μg/kg/min.

## Discusión

In the present study, we found that only 25% of donor offers were accepted, predominantly involving young male donors, with non-standard risk donor status being the leading cause of rejection.

The high 75% rejection rate observed in this study reflects a concerning reality that substantially compromises organ availability for transplantation in Ecuador. This figure mirrors trends reported in other international settings, where offer decline rates are also considerable; in the United Kingdom, a rejection rate of 76.8% was reported in 2023 ^15^. In the United States, data from the Donor Heart Study conducted by Stanford University and from the Scientific Registry of Transplant Recipients reported rejection rates of 40.2% and 54.5%, respectively ^16^^,^^17^.

Consistent with previous studies, male donors, younger age, and blood group O were associated with a higher likelihood of acceptance; similar findings have been reported by a study conducted at the Fundación Cardioinfantil in Bogotá, Colombia ^18^. In our cohort, the main rejection factors were related to intrinsic donor characteristics, such as advanced age and comorbidities (39.5%). In the analysis by Asemota *et al.* using UK data, rejections were primarily due to organ-related factors (47.6%), including cardiac dysfunction, unsuitable donor size, ABO incompatibility, organs deemed unsuitable for transplantation, infection, malignancy, anatomical abnormalities, prolonged ischaemia times, positive crossmatch, and fatty infiltration of the graft ^15^. In the United States, among 7104 hearts declined for implantation, 25.1% were rejected due to poor graft function, followed by 14.0% due to donor medical history ^19^.

Emerging evidence, however, supports the safe use of hearts from donors previously considered high-risk or non-standard ^20^^,^^21^. The predominance of non-standard risk donors as the main reason for rejection in this study (39.5%) reflects conservative selection criteria that may unnecessarily limit the utilisation of viable organs. The shift towards expanded donor criteria has demonstrated acceptable outcomes in multiple series ^21^^,^^22^. Wang et al. reported that donors meeting expanded criteria, including age ≥50 years, prolonged ischaemia time, and mild ventricular dysfunction, can be successfully used with outcomes comparable to those of standard donors ^23^. Furthermore, Bakhtiyar *et al.* showed that heart transplants using expanded-criteria donor allografts at high-volume centres provide a significant survival benefit ^24^. It is estimated that increased use of expanded-criteria organs, in appropriate clinical contexts, does not adversely affect overall patient outcomes in high-volume centres ^24^.

In this cohort, logistical issues emerged as the second leading cause of rejection (30.6%) of cardiac offers, a particularly relevant finding given their potential modifiability. A progressive increase in logistics-related rejections was observed, rising from 0% in 2021 to over 40% in 2024-2025, mainly related to air transport between regions. These figures differ in magnitude from those reported in the United Kingdom, where insufficient logistical support leads to avoidable rejections ^15^, but not to the extent observed in Ecuador. In the UK, logistical factors accounted for only 1% of rejections, primarily due to loss of fast-track offers (0.2%), lack of transport availability (0.4%), unavailability of procurement teams (0.1%), and hospital bed shortages (0.1%) ^15^. Centralisation of complex procedures in specialised centres, together with structured donor, team, and organ transfer protocols, can markedly reduce losses due to operational constraints.

In Ecuador, the availability of state-owned aircraft for procurement team mobilisation is limited. This is compounded by restrictions imposed by the National Health Services Tariff Schedule ^25^, which covers only a fraction of transport costs and does not specify whether private services may be used, effectively limiting coverage to commercial flights. Although INDOT maintains agreements with national airlines ^26^^,^^27^, these transfers are subject to significant operational constraints, including fixed schedules, unexpected flight changes, and limited cabin space. Under these conditions, the use of commercial flights for cardiac procurement teams is often impractical, as cold ischaemia time would exceed the recommended limit of less than four hours ^8^. This challenge is not unique to Ecuador; in the United States, a report by the Federal Aviation Administration’s Organ Transport Working Group demonstrated that, despite the absence of regulatory barriers to in-cabin organ transport, the lack of standardised protocols and inter-airline variability can result in significant delays ^28^. Consequently, the report recommends implementing uniform air transport procedures, targeted training for flight and security personnel, operational prioritisation through advanced coordination, and the establishment of centralised notification and monitoring systems ^28^. Adoption of similar measures in Ecuador could help reduce the proportion of logistics-related rejections observed in this series.

Recent evidence indicates that advanced preservation systems, such as controlled hypothermia (Paragonix SherpaPak®) and normothermic perfusion (OCS™ Heart), allow prolongation of ischaemia time and enable the use of expanded-criteria donors without a significant increase in primary graft failure or reduction in survival ^29^^,^^30^. However, their implementation within the public health system entails higher operational costs and requires regulatory processes that have yet to be established. Moreover, controlled donation after circulatory death (DCD) protocols are not currently available in Ecuador; consequently, all offers analysed in this study corresponded to donation after brain death.

Our findings provide local evidence to support the optimisation of national programmes and the reduction of waiting-list mortality. Donor hearts rejected in this series were not utilised by other centres due to nationwide logistical limitations. Although some grafts could potentially be allocated for valvular homografts, this alternative is infrequently used and depends on the operational and logistical capacity of the country’s two tissue banks.

Study limitations include its retrospective design and the evaluation of a single transplant centre. In addition, the lack of detailed comparative data from other national centres limits the generalisability of the findings.

In conclusion, this study documents a high 75% rejection rate of cardiac donor offers directed to Clínica Guayaquil, highlighting a substantial underutilisation of potentially viable organs that compromises access to heart transplantation in Ecuador. 

The identification of non-standard risk donors as the primary cause of rejection suggests the need for a detailed reassessment of acceptance criteria towards more permissive approaches, supported by international evidence demonstrating satisfactory outcomes with expanded-criteria donors. Logistical issues constitute the second leading cause of rejection and represent the modifiable factor with the greatest impact identified in this study. Optimising transport protocols, interhospital communication, and organ preservation strategies emerges as an immediate opportunity to substantially increase acceptance rates without altering medical selection criteria. Addressing these operational deficiencies could translate into a significant increase in the number of transplants performed annually.

Future research should evaluate the impact of targeted interventions on organ utilisation rates in the country, as well as the development of operational models to optimise organ allocation at the national level.
